# Prevalence of solid tumors and lymphoma in the Brazilian Sjögren’s Disease Registry (BRAS): an important concern for clinical practice

**DOI:** 10.1186/s42358-025-00470-7

**Published:** 2025-08-20

**Authors:** Virginia Fernandes Moça Trevisani, Fabiola Reis de Oliveira, Sandra Gofinet Pasoto, Laura Caldas, Roberta de Almeida Pernambuco, Alisson Pugliesi, Leandro Augusto Tanure, Juliana Markus, Maria Lúcia Lemos Lopes, Aysa César Pinheiro, Vanessa Hax, Aiessa Zanchett Fedrigo, Sandra Lúcia Euzébio Ribeiro, Karina Gatz Capobianco, Giovanna Sant’Ana Petterle, Diego Ustárroz Cantali, Rafael Coradin, Gustavo Pafume de Sá, Ketty Lisie Libardi Machado, Bianca Rosa, Raíssa Dudienas Domingues Pereira, Samira Tatiyama Miyamoto, Nelson Carvas Junior, Valéria Valim

**Affiliations:** 1 Universidade Federal de São Paulo, São Paulo, SP Brazil; 2 Universidade de Santo Amaro, UNISA, São Paulo, SP Brazil; 3 Hospital das Clínicas da Faculdade de Medicina de Ribeirão Preto da USP, Ribeirão Preto, SP Brazil; 4 Hospital das Clínicas HCFMUSP, Faculdade de Medicina, Universidade de São Paulo, São Paulo, SP Brazil; 5 Instituto de Assistência Médica ao Servidor Público Estadual, São Paulo, SP Brazil; 6 Hospital das Clínicas da Universidade Estadual de Campinas, Campinas, SP Brazil; 7 Hospital das Clínicas da Universidade Federal de Minas Gerais, Belo Horizonte, MG Brazil; 8 Hospital de Clínicas, Faculdade de Medicina da Universidade Federal de Uberlândia, Uberlândia, MG Brazil; 9 Irmandade da Santa Casa de Misericórdia de Porto Alegre, Universidade Federal de Ciências da Saúde de Porto Alegre, Porto Alegre, RS Brazil; 10 Hospital das Clínicas da Universidade Federal de Pernambuco, Recife, PE Brazil; 11 Hospital de Clínicas de Porto Alegre da Universidade Federal do Rio Grande do Sul, Porto Alegre, RS Brazil; 12 Hospital Universitário Evangélico Mackenzie, Curitiba, PR Brazil; 13 Universidade Federal do Amazonas, Manaus, AM Brazil; 14 Hospital Moinhos de Vento, Porto Alegre, RS Brazil; 15 Hospital Universitário Júlio Müller, Universidade Federal de Mato Grosso, Cuiabá, MT Brazil; 16 Hospital São Lucas, Pontifícia Universidade Católica do Rio Grande do Sul, Avenida Ipiranga, Porto Alegre, RS Brazil; 17 Hospital Universitário Cassiano Antônio Moraes, Universidade Federal do Espírito Santo, Vitória, ES Brazil; 18 Departamento de Educação Integrada em Saúde, Universidade Federal do Espírito Santo, Vitória, ES Brazil; 19 Universidade São Paulo, UNIP, São Paulo, SP Brazil; 20 Disciplina de Emergência e Medicina Baseada em Evidência, Departamento de Reumatologia na Universidade de Santo Amaro, Universidade Federal de São Paulo, Rua Botucatu, 744, São Paulo, SP 04023-900 Brazil

**Keywords:** Sjögren’s syndrome, Sjögren’s disease, Cancer, Lymphoma, Solid neoplasia

## Abstract

**Background:**

About 5–10% of patients with Sjögren’s Disease (SjD) will develop non-Hodgkin’s lymphoma, with a 16 to 44 increased risk compared to the general population. The relationship between SjD and other types of cancer is poorly described in the literature.

**Aim:**

To characterize patients with SjD from the Brazilian Sjögren’s Disease Registry (BRAS) who developed hematological and solid neoplasms, analyzing the organ primarily involved, subtypes, occurrence of metastatic disease, timing of diagnosis and correlation with demographic, serologic aspects, and labial salivary gland inflammation.

**Methods:**

Among consecutive patients included in the Brazilian Sjögren’s Disease Registry (BRAS) and fulfilling the 2002 or 2016 classification criteria for SjD, those who developed neoplasia were retrospectively identified and categorized according to the National Cancer Institute (Ministry of Health, Brazil). RESULTS: 1,010 patients were included; of them, 975 were women (96.5%), with an average age of 55.6 ± 13.6 years. Disease duration was 11.9 ± 7.9 years. We found that 114 out of 1,010 patients (11.3%) had cancer, with a higher prevalence in those over 55 years old (*p* < 0.001). The most prevalent malignancies were skin cancer [24/858 (2.7%)], breast cancer [27/1010 (2.6%)], lymphoma [15/1010 (1.5%)], and thyroid cancer [8/1010 (0.8%)]. Further, 0.9% of patients had more than one type of cancer. Cancer diagnosis followed SjD diagnosis in 66.7% of lymphoma cases, 53.6% of other malignancies, and 75.0% of skin cancer. The presence of cancer was associated with age (OR 1.04). In the case of skin cancer, the duration of SjD was an additional risk factor (OR 1.08). For thyroid cancer, inflammation of the labial minor salivary glands was identified as an associated risk factor (OR 10.1).

**Conclusions:**

Other types of cancer were more prevalent than lymphoma in our population of SjD patients. Breast neoplasia emerged as the most prevalent after skin cancer, and thyroid cancer was the second most prevalent solid neoplasia. Aging, disease duration and labial salivary gland inflammation were associated risk factors. Prospective cohort studies are essential for assessing other risk factors for cancer development in SjD patients.

## Background

Sjögren’s syndrome, recently referred to as Sjögren’s disease (SjD) [1], is a chronic immune-mediated condition mainly characterized by lymphocytic inflammatory injury of the salivary and lacrimal glands, causing tissue damage and glandular dysfunction. Furthermore, multiple organs may be involved, leading to a broad clinical spectrum that includes fatigue, fever, weight loss, polyarthralgia, polyarthritis, palpable purpura, bronchiolitis and interstitial lung disease, tubulointerstitial nephritis, glomerulonephritis and peripheral/central neuropathies [2]. The global prevalence of SjD is estimated at 60.82 [95% CI (Confidence Interval) 43.69–77.94] patients per 100,000 inhabitants, with variations in different countries [3]. In Brazil, the prevalence of SjD is approximately 0.17% [4]. This disease mainly affects women aged 40–60 years [5].

The etiopathogenesis of SjD is not fully understood, but there is evidence of the participation of genetic, hormonal, environmental and epigenetic factors, leading to a deregulation of the immune system [2]. Multiple susceptibility alleles are involved, demonstrating a polygenic pattern. In this regard, type I interferon (IFN) genes [e.g., STAT4 (Signal Transducer and Activator of Transcription 4) and IRF5 (IFN Regulatory Factor 5)], known as the IFN-α gene signature, can lead to anomalous activation of B lymphocytes [6, 7]. The Human Leukocyte Antigen (HLA) complex (e.g., HLA-DRB1*03:01, HLA-DQA1 and HLA-DQB1) is also involved [6, 7]. Associations with other genes, such as CXCR5 (C-X-C Motif Chemokine Receptor 5), NF-kB (Nuclear Factor kB) and BLK (B Lymphoid Tyrosine Kinase), related to the proliferation of B cells, show the great complexity of genetic factors in SjD [6, 7]. The hormonal aspects include effects of sex steroids on the ocular surface and glandular epithelial cells, in addition to contributing to immunological imbalance [8]. Epigenetic factors and environmental “triggers”, such as infectious agents, mainly the Epstein-Barr virus (EBV) [9], can precipitate the onset of disease in the susceptible host. Cells of the innate and adaptive immune system play an invaluable part in the pathogenesis of SjD [10]. Cellular interactions can amplify and perpetuate the chronic inflammatory process, leading to tissue damage and glandular dysfunction. Antigen-presenting cells express the IFN-induced gene signature, stimulating the production of pro-inflammatory cytokines. Salivary gland epithelial cells play a central role in the pathogenic mechanisms of SjD, interacting with T and B lymphocytes through the presentation of self-antigens as a result of apoptosis, as well as producing type I IFN and chemokines, such as B-cell Activating Factor (BAFF) [10]. A notable feature of SjD is the hyperactivation of B cells, with the production of autoantibodies, mainly anti-Ro/SS-A and anti-La/SS-B, as well as a high risk for developing non-Hodgkin’s lymphoma, which has been reported around 5% of patients [11].

In this regard, the Standardized Incidence Ratios (SIR) of non-Hodgkin’s lymphoma are 7.4 [95% CI 3.3–17.0], 3.9 (95% CI 2.5–5.9) and 18.8 (95% CI 9.5–37.3) for systemic lupus erythematosus (SLE), rheumatoid arthritis (RA) and SjD, respectively [12]. Recently, it was also observed that SjD patients are at increased risk of cancer in general, including other hematological malignancies and solid tumors [13, 14]. On the other hand, there are few studies showing the relationship between solid malignancies and SjD. A systematic review and meta-analysis including 47,607 participants demonstrated that SjD was significantly associated with increased overall cancer risk (SIR 2.17, 95% CI 1.57-3.00), hematological malignancies (SIR 11.55, 95% CI 4.32–30.90), including non-Hodgkin’s lymphoma (SIR 13.71, 95% CI 8.83–21.29), and solid tumors (SIR 1.39, 95% CI 0.90–2.13) [14].

There are no studies in the Brazilian population on the prevalence of cancer in SjD patients. Therefore, the present registry study aims to evaluate the prevalence of hematological and solid neoplasms in the cohort of the Brazilian Sjögren´s Disease Registry (BRAS), and to provide a real-world understanding of the epidemiological aspects and outcomes of individuals with SjD. Additionally, risk factors for development of neoplasms and the profile of the most common malignancies in these patients are assessed.

## Methods

### Patients

The Brazilian Sjögren’s Disease Registry (BRAS) is a national, multicenter registry, approved in 2018 according to document 2.930.715/2018 of the ethics committee and registered with number 99071018.3.1001.5071. To be included in the registry, patients must meet the classification criteria of 2002 or 2016 [15, 16]. The exclusion criteria were as follows: patients with SjD associated with other systemic immune-mediated diseases, patients with active hepatitis C virus infection, HIV, amyloidosis, sarcoidosis, IgG4-related disease and pre-existing irradiation in the neck region. Fifteen reference centers in different areas of the country included patients. For the analysis of this retrospective cohort, 1,010 patients included from 2019 to 2021 were examined for the development of neoplasm, analyzing the organ primarily involved, cancer subtypes, occurrence of metastasis, more than one neoplasm in the same individual, timing of diagnosis and correlation with demographic, serologic aspects, and labial salivary gland inflammation. For skin cancers, data were available only on 858 patients. The medical team that accompanies the patients completed the data retrospectively in the database during patient consultations. If necessary, the patients were called for an evaluation and, if this was not possible, telephone contact was made to complete the required information. Biopsy results and serologic aspects were collected from the patient’s clinical files.

Patients were actively asked about the history of neoplasms and their characteristics. The initial analysis provided a descriptive overview of the quantitative variables of age and diagnosis time. The descriptive results are presented as median, mean and standard deviation, coefficient of variation (CV), minimum and maximum values and quartiles [1st quartile (Q1) shows the distribution up to 25% of the sample, and the 3rd quartile (Q3) shows the distribution up to 75% of the sample]. The qualitative variables are presented as relative frequencies (prevalence). We used non-parametric statistical tests, as we tested the normality of the main quantitative outcome variables using the Shapiro-Wilk test and concluded that there was no assured normal distribution. We utilized logistic regression analysis to calculate the likelihood of each disease testing positive. Initially, we ran the model using univariate analysis, examining one variable at a time. Subsequently, we conducted multivariate analysis, considering all the variables together to obtain a comprehensive understanding of the relationships. A significance level of 0.05 (5%) was set. All confidence intervals were constructed with 95% statistical confidence.

## Results

Among the 1,010 consecutive patients included in the BRAS, a significant majority were women, accounting for 96.5% of the total. The patient population also included 523 white individuals, representing 52% of the total, with an average age of 55.6 ± 13.6 years. The average time of illness (first symptom) was 11.9 ± 7.9 years, and the average time of diagnosis of SjD was 8.0 ± 6.2 years. The average time to cancer diagnosis was 8.4 ± 9.0 years (Table 1). Notably, 937 patients (93%) experienced ocular symptoms, and 942 (93.7%) had oral symptoms, highlighting the prevalence and impact of these manifestations. Additionally, 786 (79.6%) were positive for anti-Ro/SSA antibodies and 414 (44.3%) for anti-La/SSB antibodies (Table 2).

**Table 1:** Baseline characteristics of 1010 patients with sjögren’s disease

		Mean	SD
Age (years)		55.6	13.6
Disease duration (years)		11.9	7.9
Time to Sjögren’s Disease diagnosis (years)		8.0	6.2
Time to cancer diagnosis (years)		8.4	9.0
Time to lymphoma diagnosis (years)		8.3	9.7
Time to skin cancer diagnosis (years)		8.9	9.8
		**N**	**%**
Ethnicity	White	523	52.0
	Mixed race	308	30.6
	Black-African American	77	7.7
	Asian	10	1.0
	Indigenous	2	0.2
	NR	86	8.5
Gender	Female	975	96.5
Marital status	Married	489	56.1
	Divorced	189	21.7
	Single	164	18.8
	NR	29	3.3
Employment status	Employed	322	37.6
	Retired	267	31.2
	Housework	208	24.3
	Unemployed	49	5.7
	Study	11	1.3

NR – not reported

SD – standard deviation

**Table 2:** Clinical data of 1010 patients with sjögren’s disease

	Yes		No		
	*N*	%	*N*	%	Total
Ocular symptoms	937	93.1	69	6.9	1006
Oral symptoms	942	93.7	63	6.3	1005
Altered ocular signs	705	74.8	237	25.2	942
Schirmer’s I test	636	71.5	253	28.5	889
Ocular dye score	272	66.2	139	33.8	441
Focal lymphocytic sialadenitis	574	86.3	91	13.7	665
Salivary gland involvement	620	73.0	229	27.0	849
Unstimulated whole salivary flow	465	78.7	126	21.3	591
Parotid sialography	12	46.2	14	53.8	26
Salivary scintigraphy	242	90.0	27	10.0	271
Positive anti-Ro (SSA)	786	79.6	202	20.4	988
Positive anti-La (SSB)	414	44.3	521	55.7	935

Out of 1,010 SjD patients, 114 developed cancers, which represents 11.3% of the total, and it was significantly more frequent in patients over 55 years (*p* < 0.001). The most prevalent malignancies were skin cancer [24/858 (2.7%)], breast cancer [27/1010 (2.6%)], lymphoma [15/1010 (1.5%)], and thyroid cancer [8/1010 (0.8%)]. Additionally, 0.9% of patients had more than one type of cancer. Of the 24 patients with skin cancer, basal cell carcinoma was the most prevalent [14 (59%)], followed by melanoma [5 (21%)]. Lymphoma was the third most prevalent cancer after breast cancer in our population. B-proliferative neoplasms were the major hematological cancer subtype [14 (73%)]. Thyroid cancer was the fourth most prevalent [8 (0.8%)], followed by ovarian cancer in 4 cases (0.5%) (Table 3).

**Table 3:** Distribution and frequency of cancer types

		*N*	%
Cancer		114/1010	11.3
Skin cancer		24/858	2.7
	Basal cell carcinoma	14	1.6
	Squamous cell carcinoma	4	0.4
	Melanoma	5	0.5
	Vulval intraepithelial neoplasia	1	0.1
Lymphoma		15/1010	1.5
	MALT lymphoma	5	0.5
	Diffuse large B cell lymphoma	3	0.3
	Nodal marginal zone lymphoma	2	0.2
	Other non-Hodgkin lymphoma	2	0.2
	Lymphoplasmacytic lymphoma	1	0.1
	Hodgkin lymphoma	2	0.2
	Multiple myeloma	1	0.1
Other hematological cancer		3/1010	0.3
	Polycythemia vera	1	0.1
	Myelodysplastic syndrome	1	0.1
	Chronic myelomonocytic leukemia	1	0.1
Other cancers		71/1010	7.0
	Breast	27	2.6
	Uterine cervix	3	0.3
	Ovarian cancer	5	0.5
	Endometrial carcinoma	2	0.2
	Prostate	2	0.2
	Thyroid	8	0.8
	Parathyroid	1	0.1
	Parotid (Warthin tumor)	2	0.2
	Parotid (other)	1	0.1
	Lung	2	0.2
	Stomach	2	0.2
	Liver	1	0.1
	Pancreas	1	0.1
	Gallbladder	1	0.1
	Urothelial carcinoma	3	0.3
	Anorectal cancer	3	0.3
	Colon cancer (sigmoid)	1	0.1
	Glioblastoma	1	0.1
	Meningioma	1	0.1
	Pituitary adenoma	1	0.1
	Giant cell tumor of bone	1	0.1
	Eye (squamous cell carcinoma)	2	0.2
More than one cancer type		9/1010	0.9

Table 4 shows that the diagnosis of cancer followed the diagnosis of SjD in 66.7% of lymphoma, 53.6% of other neoplasms, and 75.0% of skin cancers. We observed that the diagnosis of cancer after the SjD diagnosis was statistically more frequent than the diagnosis of cancer prior to or concomitant with SjD.

**Table 4:** Time between sjögren’s disease diagnosis and cancer diagnosis

	Before		Concomitant		After	
	*N*	%	*N*	%	*N*	%
Cancer	23	33.3	9	13.0	37	53.6
Lymphoma	2	13.3	3	20.0	10	66.7
Skin cancer	2	25.0	0	0.0	12	75.0

The main risk factor for developing cancer was the age (OR 1.04). This was also observed in patients who developed breast cancer and skin cancer. In the case of skin cancer, the duration of the disease was also found to be an associated risk factor (OR 1.08). For thyroid cancer, which was the third most prevalent solid tumor, inflammation of the salivary glands (labial minor salivary gland biopsy positivity) was identified as an important associated risk factor (OR 10.1) (Tables 5 and 6).

**Table 5:** Univariate and multivariate logistic regression of risk factors for cancer

	Variables	Coefficient (B)	P	Odds Ratio	
				OR	95% CI
Univariate	Age	0.042	**< 0.001**	**1.04**	**1.03–1.06**
	Disease duration	0.033	**0.008**	**1.03**	**1.01–1.06**
	Time to SjD diagnosis	0.031	**0.039**	**1.03**	**1.00–1.06**
	Gender (Female)	0.176	0.775	1.19	0.36–3.96
	Ethnicity (white)	0.416	0.062	1.52	0.98–2.35
	Positive salivary gland biopsy	-0.162	0.671	0.85	0.40–1.80
	Anti-SSA	-0.618	**0.008**	**0.54**	**0.34–0.85**
	Anti-SSB	-0.312	0.159	0.73	0.47–1.13
Multivariate	Age	0.035	**0.006**	**1.04**	**1.01–1.06**
	Disease duration	0.008	0.721	1.01	0.96–1.05
	Time to SjD diagnosis	0.025	0.448	1.03	0.96–1.10
	Gender (Female)	-0.120	0.879	0.89	0.19–4.16
	Ethnicity (white)	-0.249	0.423	0.78	0.42–1.43
	Positive salivary gland biopsy	-0.441	0.294	0.64	0.28–1.47
	Anti-SSA	-0.494	0.176	0.61	0.30–1.25
	Anti-SSB	-0.296	0.437	0.74	0.35–1.57

SjD – Sjögren’s Disease

CI – Confidence interval

OR – Odds ratio

**Table 6:** Simple and multivariate analysis of risk factors for the most frequent types of cancer

	Variables	Skin cancer			Breast cancer			Lymphoma			Thyroid cancer		
		OR	95% CI	P	OR	95% CI	P	OR	95% CI	P	OR	95% CI	P
Simple	Age	**1.08**	**1.04–1.12**	**< 0.001**	**1.04**	**1.01–1.07**	**0.007**	1.01	0.97–1.05	0.589	1.01	0.96–1.07	0.69
	Disease duration	**1.08**	**1.04–1.12**	**< 0.001**	1.00	0.98–1.01	0.71	1.03	0.97–1.09	0.315	0.99	0.96–1.02	0.56
	Positive salivary gland biopsy	0.41	0.10–1.61	0.201	0.99	0.51–1.92	0.98	1.43	0.18–11.45	0.734	**6.16**	**1.99–22.3**	**0.002**
	Anti-SSA	0.43	0.17–1.05	0.063	0.85	0.37–2.22	0.73	0.70	0.22–2.23	0.549	0.74	0.17–4.41	0.71
	Anti-SSB	0.54	0.20–1.42	0.210	1.86	0.86–4.16	0.12	0.34	0.09–1.22	0.098	1.68	0.37–8.57	0.50
Multiple	Age	**1.12**	**1.04–1.21**	**0.003**	**1.04**	**1.01–1.08**	**0.018**	1.02	0.96–1.07	0.517	0.98	0.93–1.04	0.59
	Disease duration	**1.08**	**1.01–1.15**	**0.028**	0.99	0.97–1.01	0.38	0.91	0.76–1.11	0.356	0.98	0.94–1.02	0.49
	Positive salivary gland biopsy	0.23	0.04–1.26	0.091	0.84	0.40–1.73	0.63	0.96	0.11–8.43	0.970	**10.1**	**2.84–46.4**	**< 0.001**
	Anti-SSA	0.31	0.05–1.80	0.191	1.45	0.40–6.66	0.60	0.86	0.20–3.72	0.842	0.37	0.04–6.92	0.42
	Anti-SSB	0.34	0.03–3.89	0.385	1.87	0.71–5.38	0.22	0.16	0.02–1.47	0.104	2.60	0.40–24.9	0.34

CI – Confidence interval

OR – Odds ratio

In our registry, we found that B-cell lymphoma had no relationship in the multiple analyses with age, duration of disease, or the presence of anti-Ro/SSA and anti-La/SSB antibodies (Table 6).

Regarding the prognosis, 18 out of 24 (90%) with skin cancer, 13 out of 16 (81.2%) with B-cell neoplasms (lymphoma and multiple myeloma), and 51 (81%) with other cancers progressed to cure, 4 (6.3%) patients had metastases, 4 (6.3%) relapsed, and 4 (6.3%) died.

## Discussion

This study was the first to assess the prevalence of cancer in a Brazilian population with SjD, and it provides relevant information for patient follow-up. The profile of the most prevalent malignancies, with emphasis on lymphoma (3rd place) and thyroid cancer (4th place), appears to be different from that observed in the general Brazilian population. Furthermore, we observed that the diagnosis of cancer after the SjD diagnosis was statistically more frequent than the diagnosis of cancer prior to or concomitant with SjD, and that thyroid cancer was associated with greater inflammation in the salivary glands, suggesting a role for the underlying autoimmune disease as a risk factor for the development of this neoplasm.

The main advantage of the present study was the inclusion of participants recruited from centers located in all macro-regions of the country, thus reflecting the Brazilian patient population. On the other hand, the main limitations were the retrospective study design, and the lack of some disease parameters associated with a higher risk of developing lymphoma, such as the high degree of systemic activity, presence of cryoglobulins, decreased levels of complement fractions C3 and C4, monoclonal gammopathy, neutropenia and lymphopenia [11, 17–20].

Furthermore, our data were derived from a national, multicenter cohort of patients with Sjögren’s Disease, collected through the Brazilian Society of Rheumatology. Due to the observational nature and specific scope of the cohort, it was not feasible to include an internal control group composed of individuals without Sjögren’s Disease, followed over the same period and under similar conditions. For this reason, we used national cancer incidence estimates published by the Brazilian National Cancer Institute (INCA) [21] as an external reference to discuss our results. However, we recognize that the use of crude frequencies without adjustment may limit the strength of the conclusions regarding whether cancer prevalence in patients with Sjögren’s Disease is genuinely different from that in the general population.

In the Brazilian female population, the malignancies with the highest incidences (except non-melanoma skin cancer) in 2023 were: 1st breast, 2nd colon and rectum, 3rd cervix, 4th trachea, bronchus and lung, 5th thyroid gland, 6th stomach, 7th body of the uterus, 8th ovary, 9th pancreas and 10th non-Hodgkin lymphoma [21]. In contrast, in our predominantly female SjD population, the most prevalent cancer types (except skin) were breast cancer (first), lymphoma (second), and thyroid cancer (third). These apparent differences in the profile of the malignancies’ prevalence provide relevant information for the clinical follow-up of SjD patients. Nevertheless, we were unable to perform direct age-standardized comparisons with INCA data due to differences in data granularity and availability. Such an approach would enhance comparability. Thus, future population-based studies with matched controls and standardized comparisons are necessary to validate our findings.

The association between DSj and lymphoma is well known. A recent systematic review and meta-analysis study including 50,308 participants from articles published from 1997 to 2023 showed an SIR of non-Hodgkin’s lymphoma of 8.78 (95% CI 5.51, 13.99) [22]. The prevalence of lymphoma in SjD is estimated from 4 to 11% [11, 17, 23]. In our registry, we found that B-cell lymphoma had a lower prevalence (1.5%) compared to other cohorts worldwide and showed no relationship in the multiple analyses with age, duration of disease, or the presence of anti-Ro/SSA and anti-La/SSB antibodies. In this regard, a single-center study in Brazil including 97 SjD patients reported a 3% prevalence of lymphoma [24]. A cross-sectional study of SjD patients followed at the University of Florida Health Shands Hospital showed a prevalence of lymphoma of 2.6% that was also lower than that of previous studies [25]. Some hypotheses may theoretically explain this finding, such as differences in the genetic background of the populations evaluated, short time of follow-up, as well as a lower degree of severity of the underlying autoimmune disease [25]. However, future studies comparing the prevalence of lymphoma in different SjD populations may clarify these hypotheses [26, 27].

Solid neoplasms are also relevant comorbidities in SjD patients [28]. A systematic review and meta-analysis including 25 studies, 47,607 individuals, with a follow-up of 452,468 person-years, demonstrated an increased risk of solid tumors (SIR 1.39, 95% CI 0.90–2.13), including lung cancer (SIR 1.55, 95% CI 1.29–1.85), thyroid cancer (SIR 2.05, 95% CI 1.20–3.48), non-melanoma skin cancer (SIR 1.71, 95% CI 1.08–2.72), kidney/urinary tract cancer (SIR 1.36, 95% CI 1.02–1.81), liver cancer (SIR 1.70, 95% CI 1.13–2.57), and prostate cancer (SIR 1.50, 95% CI 1.02–2.22) in SjD patients [14].

A nationwide population-based retrospective study from the French health insurance database evaluated hospitalized patients with new-onset SjD from 2011 to 2018 compared to age- and sex-matched hospitalized controls [29]. Among 25,661 hospitalized SjD patients versus 252,543 matched patients (mean follow-up 4.0 years), the incidence of thyroid cancer was higher [aHR (adjusted Hazard Ratio) 1.7 (1.1–2.8)], while the incidences of bladder and breast cancer were lower [aHR 0.58 (0.37–0.89) and 0.60 (0.49–0.74), respectively] in SjD patients [29]. Other studies also confirm the higher frequency and risk of thyroid cancer in SjD, including the Spanish cohort (GEAS-SS Study Group) [30] and data from Korean Health Insurance Claims Database [31]. Hashimoto’s thyroiditis, which is associated with thyroid cancer [32, 33], is common in SjD patients [34]. Interestingly, thyroid cancer in our population was associated with positivity in labial minor salivary gland biopsy, suggesting a role for underlying disease-related inflammation in the pathophysiology of this sort of cancer. Future studies should evaluate whether comorbidity with Hashimoto’s thyroiditis increases the risk of thyroid cancer in SjD patients.

Regarding breast cancer, a recent systematic review and meta-analysis suggested geographical differences in the association with SjD. In European populations, a lower risk of breast cancer was reported, but it was higher in Asia and Argentina [35]. Future studies are needed to confirm these findings. In our sample, breast cancer was the second most prevalent.

## Conclusions

In conclusion, the profile of most prevalent tumors in SjD patients apparently differed from the general population, with emphasis on lymphoma (3rd place) and thyroid cancer (4th place). Our study has limitations due to its retrospective design, and it was not feasible to include an internal control group composed of individuals without SjD. Therefore, other prospective cohort studies are essential for assessing risk for cancer development in SjD patients.

## Data Availability

No datasets were generated or analysed during the current study.

## Abbreviations

aHR: Adjusted Hazard Ratio
BAFF: B-cell Activating Factor
BLK: B Lymphoid Tyrosine Kinase
BRAS: Brazilian Registry of Sjögren’s Disease
CV: Coefficient of variation
CXCR5: C-X-C Motif Chemokine Receptor 5
EBV: Epstein-Barr virus
HLA: Human Leukocyte Antigen
IFN: Interferon
IRF5: Interferon Regulatory Factor 5
NF-kB: Nuclear Factor kB
RA: Rheumatoid arthritis
SIR: Standardized Incidence Ratios
SjD: Sjögren’s disease
SLE: Systemic lupus erythematosus
STAT4: Signal Transducer and Activator of Transcription 4
